# Ventilation methods to prevent the spread of airborne pathogens from teachers to students

**DOI:** 10.1007/s44189-023-00024-w

**Published:** 2023-03-29

**Authors:** Chang Heon Cheong, Seok-Ho Hwang, Beungyong Park

**Affiliations:** 1 School of Architectural Engineering, Gyeongsang National University, Jinju-Si, South Korea; 2 School of Architecture, Kyungnam University, Changwon-si, South Korea; 3 Department of Building and Plant Engineering, Hanbat National University, Daejeon, South Korea

**Keywords:** School classroom, Ventilation, Airborne infection, CFD simulation, Source control strategy

## Abstract

This study investigates different ventilation systems to prevent the spread of airborne pathogens from teachers in an elementary school classroom setting. The analyzed systems include a general mechanical ventilation system and a hybrid ventilation system. The hybrid ventilation system used a combination of natural ventilation, general mechanical ventilation, and local mechanical ventilation systems. For natural ventilation, wind velocities of 1.1 m/s and 0.11 m/s were considered. To analyze the patterns of the spread of airborne pathogens, the indoor airflow patterns and concentrations of airborne pathogens (passive scalar) were examined using Star-CCM + . Comparing the methods confirmed that natural ventilation was more effective than general mechanical ventilation in removing the airborne pathogens discharged from the teacher. The proposed hybrid ventilation method with combined natural and mechanical ventilation also showed promise in removing airborne pathogens. However, for natural ventilation with low wind velocity, the buoyancy effect around the occupants creates airflow vortices in the front of the classroom which spread airborne pathogens from the teacher toward the students seated in the front of the classroom. Furthermore, operating a local ventilation system close to the teacher reduced the spread of airborne pathogens that occurred under natural ventilation conditions with low wind velocity.

## Introduction

COVID-19 (coronavirus disease-19) caused by SARS-CoV-2 has had numerous damaging consequences on human society. Small particles of SARS-CoV-2 may remain suspended in the air for a long time, leading to an increased risk of spreading the infection [1]. The case of infection in a restaurant in Guangzhou, China, shows the possibility of airborne transmission of SARS-CoV-2. Research confirmed that the virus-carrying aerosol was spread over 2 m through the HVAC system [2]. Lednicky et al. [3] used VIVAS air samplers to examine whether airborne transmission of viable SARS-CoV-2 occurred in the hospital room of two COVID-19 patients. Air samples were collected 2–4.8 m from two infected patients in a hospital room, and the virus strains were analyzed to confirm that the isolated virus strain was identical to that of the admitted patient. The results of the previous studies clearly indicate that aerosols may serve as a source of viral transmission. Wei et al. reported that the outbreaks of previous severe epidemics were found to be closely related to the transmission of infectious particles between persons in indoor environments [4]. In order to satisfy the requirement of a healthy environment and thermal comfort performance of indoor ventilation system, prevention of indoor pollution is essential, especially considering of purpose of disease transmission resistance [5].

In their school environment, students are vulnerable to airborne infection as they occupy the same space over an extended length of time for classes. A classroom may host several teachers and student groups each day. If a teacher happens to be infected, the teacher can transmit the virus to several class groups. Therefore, it is necessary to investigate the viruses transmitted from a teacher in a classroom setting and potential mitigation strategies.

Mirzaie et al. [6] studied the spreading of droplets containing the COVID-19 viruses expelled by coughing of an infective person(teacher) standing in a partitioned and non-partitioned classroom. The turbulent airflow was simulated using the k-ε model that the effect ventilation airflow speeds (3 m/s, 5 m/s, 7 m/s) on the dispersion of droplets of different sizes. Park et al. [7] studied the filed measurements to analyze the natural ventilation performance in a school building according to the window opening rates and positions. The results showed that the average ventilation rates were measured at 6.51 times/h for 15% window opening. It also reported that the infection probability is less than 1% when a mask is worn and more than 15% of the windows are open with cross-ventilation. However, there is no specification ventilation strategy research with teachers and student infections.

This study analyzed different ventilation methods to prevent the indoor spread of the virus discharged into the air from the infected person (i.e., teacher) during a class period in elementary school classrooms. The most optimal ventilation method was determined by comparing different ventilation conditions including natural ventilation, general mechanical ventilation, and local ventilation. Based on the analyses, we aim to propose an effective ventilation method to remove airborne pathogens discharged from a teacher in an elementary school classroom setting.

### Ventilation to prevent airborne infection

Natural ventilation, mechanical ventilation, and a hybrid ventilation method that combines natural ventilation and mechanical ventilation are investigated. The US Environmental Protection Agency [8] has emphasized the necessity of installing mechanical ventilation systems to prevent airborne infections. The recommendation points out the need for continuous ventilation by mechanical ventilation since it is not always possible to open windows and doors for natural ventilation in a building. The American Society of Heating, Refrigerating, and Air-Conditioning Engineers has published a guideline for reducing the risks of community transmission of COVID-19 to use building mechanical and passive ventilation systems in education [9].

It is mandatory to install a mechanical ventilation system when building a new school in South Korea. Currently, ventilation standards for educational facilities in the country are defined in “Rules on Facility Standards for Buildings” by the Ministry of Land, Infrastructure, and Transport. For educational facilities, the required ventilation rate is at least 36 m^3^/person·h [10]. The dimensions of elementary school classrooms in South Korea have a standardized design, and the number of occupants per class is set at 20–22. Therefore, the required ventilation rate for a single class is 720–800 CMH (cubic meter per hour; m^3^/h). The airflow rate of the mechanical ventilation system installed for each class is approximately 800 CMH. As of 2018, a mechanical ventilation system is applied to 28,053 (33%) out of 78,953 total classrooms in South Korea, while the remaining schools resort to the natural ventilation method [11].

An increase in room ventilation rates is normally expected to decrease concentrations indoors, and such an increase is used to reduce airborne disease. Jiang et al. [12] proposed that a safe ventilation rate for eliminating airborne SARA-Co-V infection is to dilute the air emitted by a SARS patient 10,000-fold with clean air. The World Health Organization [13] suggested that a natural ventilation volume of 150 m^3^/h per person is used in environments housing infectious patients. Currently, the standard of the required ventilation rate applied aims to remove carbon dioxide discharged from occupants and volatile organic compounds (VOCs) emitted from building materials. Therefore, it is judged likely that these standards may not adequately remove airborne pathogens such as SARS-CoV-2.

In addition, in elementary schools, a general ventilation system is applied in which the air of the entire classroom is mixed to dilute the concentration of air pollutants or airborne pathogens. In general, a local ventilation system applied in the vicinity of the source of pollutant emission has the advantage of fast ventilation, but since the system is only applied to kitchens or chemical hoods, it is hardly utilized in classroom settings. There have also been few studies on the analysis of airborne infection risk with different ventilation methods applied in elementary schools in South Korea.

**Fig. 1:**
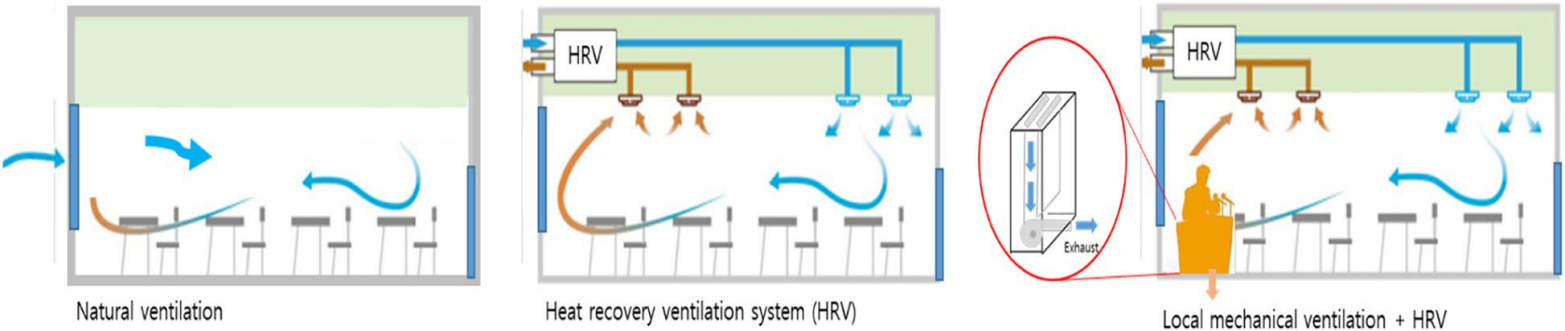
Type of ventilation system researched: natural ventilation, mechanical ventilation, and local mechanical ventilation

Therefore, in this study, the effect of removing airborne pathogens discharged from a teacher was analyzed using computational fluid dynamics (CFD) simulation for different ventilation methods. These include a mechanical ventilation system with air supply and exhaust from the ceiling which is a conventional ventilation system applied to elementary school buildings, local ventilation system, and natural ventilation. Furthermore, based on the analysis, improvements were proposed to minimize the risk of airborne pathogens discharged from a teacher in elementary school classrooms. Figure 1 shows the different types of ventilation in classrooms.

## Method

### Simulation models

A hypothetical classroom of a Korean elementary school was set as the simulation model. In this model, the infected person was the teacher who was assumed to be positioned at the front of the classroom. Students were seated in the center of the room. Therefore, in this model, airborne pathogens were set to spread from the teacher in the front of the room toward the students in the center of the room. The dimensions of the classroom were defined as 8.4 m (w) × 8.1 m (d) × 2.6 m (h). A total of five conditions were set up to analyze the spread of airborne pathogens from teacher to students under the conditions of mechanical ventilation and natural ventilation.

Case 1 described a basic general mechanical ventilation condition and the ventilation rate was set to 6.0 ACH. This corresponded to 1069 CMH which is higher than the national requirement for school classroom ventilation (21.6 CMH/ per person). In addition, 6.0 ACH (air change (m^3^) to volume of a specific space(m^3^) per hour; 1/h) also corresponds to the minimum ventilation rate condition of the negative pressure ward for the management of airborne transmission and infection [14].

Case 2 represented a condition where natural ventilation was performed by opening half of the windows in the simulation model. This reflects the realistic situation of South Korean classrooms which have sliding windows. For the airflow velocity of incoming outdoor air from the windows, data from existing research was used for reference, and the velocity was set to 1.1 m/s, which corresponded to half the average September wind velocity in Incheon (2.2 m/s) [15]. This represented a situation where airflow is introduced rapidly from the outside and where the indoor airborne pathogens are rapidly dispersed. The indoor ventilation rate was 107.4 ACH, which is an extremely high ventilation rate.

Case 3 represented the same condition as Case 2 with the windows half-open, however, the incoming airflow velocity from the window was 0.11 m/s. This corresponded to an outdoor wind velocity as low as 1/20 of the regional average wind velocity and accommodated the changes in airflow velocity of outdoor air which occur when natural ventilation is performed. Therefore, the influence of buoyancy on the indoor airflow was greater than in Case 2. In addition, the discharge of airborne pathogens was not as active compared to Case 2 due to the low indoor airflow velocity. Instead, the indoor ventilation rate was 10.7 ACH, which was higher than the ventilation rate under mechanical ventilation conditions which was 6.0 ACH.

Case 4 described a hybrid ventilation condition in which the general mechanical ventilation conditions of Case 1 and the natural ventilation conditions of Case 3 were applied simultaneously. This combined general mechanical ventilation for rapidly discharging indoor airborne pathogens in a situation where there is limited wind and natural ventilation. This condition was trial run in a real classroom during the COVID-19 pandemic in South Korea. The indoor ventilation rate was 16.7 ACH, which was equal to the sum of the mechanical ventilation rate of 6.0 ACH and the natural ventilation rate of 10.7 ACH.

Case 5 represented a condition in which the local ventilation system was installed on the teacher’s desk and operated under the condition of natural ventilation with low wind velocity, a condition corresponding to Case 3. This rapidly removed airborne pathogens discharged from the teacher before they could spread throughout the classroom. The size of the exhaust vent on the teacher’s desk was set to 0.4 m × 0.8 m, and the face velocity of the exhaust side was set to 0.1 m/s. This ventilation system without an air supply operating with air exhaust only, and the exhaust rate was 0.65 ACH. The air was supplied by the inflow of outdoor air through the windows.

**Fig. 2:**
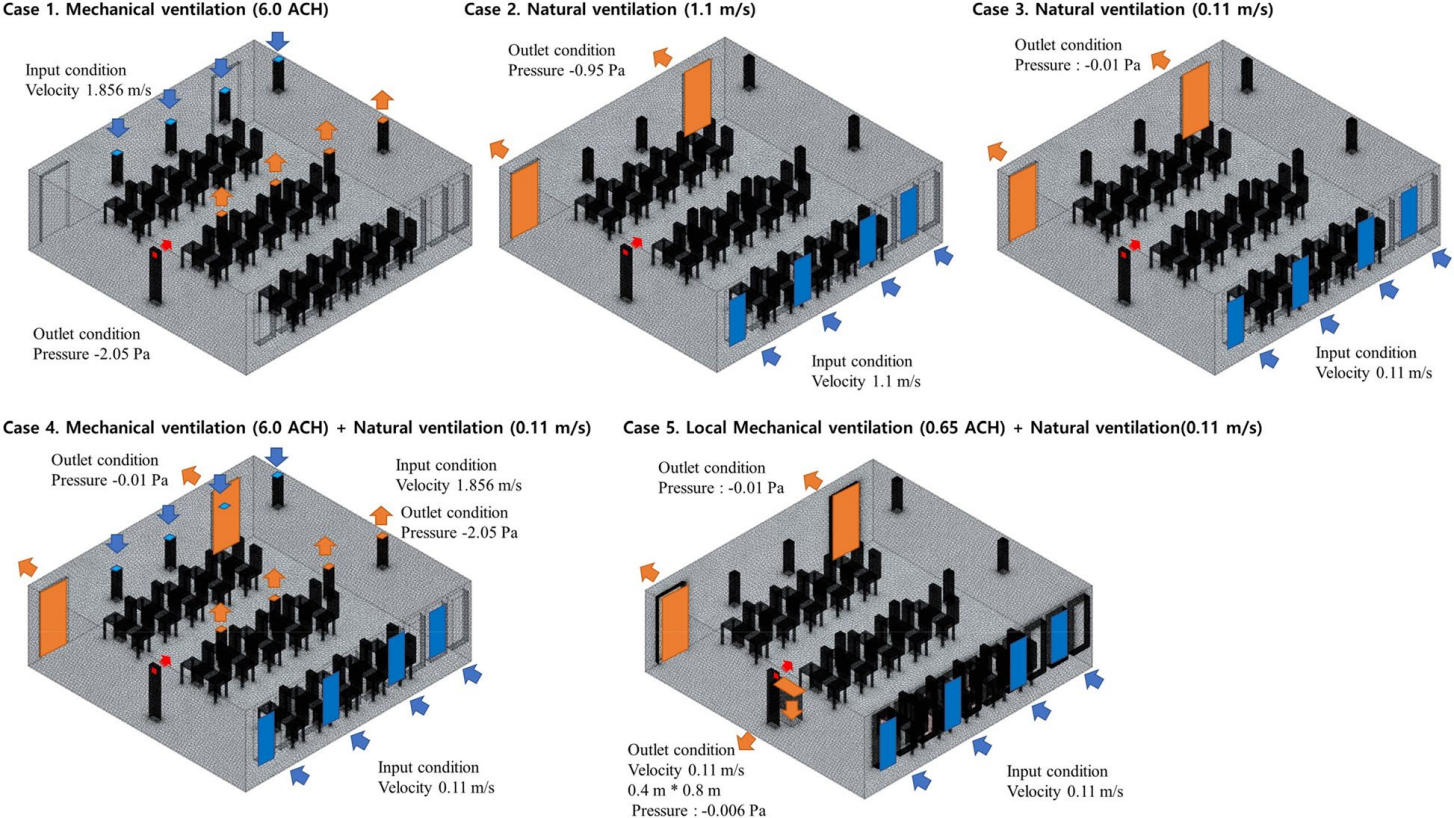
Geometric features of simulation models

The ASHRAE position document on airborne infectious diseases also recommends personalized ventilation by local exhaust and source control ventilation for use as effective measures to control and prevent disease transmission [16]. This proposed strategy is in keeping with evidence that personalized ventilation can provide better protection against airborne infection than the mixed natural ventilation. Figure 2 shows the geometric features of the simulation model for each case. Table 1 presents an overview of the simulation cases.

**Table 1:** Overview of the simulation cases

Models	Ventilation mode	Number of cells (volume mesh)
Case 1	Ambient mechanical ventilation (6.0 ACH)	16,150,067
Case 2	Natural ventilation (107.4 ACH)	7,576,531
Case 3	Natural ventilation (10.7 ACH)	
Case 4	Ambient mechanical ventilation (6.0 ACH) + natural ventilation (10.7 ACH)	
Case 5	Local mechanical ventilation (0.65 ACH) + natural ventilation (10.7 ACH)	7,659,861

### Boundary conditions

Table 2 shows the boundary conditions of the simulation model, in which analysis was performed based on a steady state. The indoor air was assumed to be an incompressible ideal gas, and the segregated flow model was implemented. The k-e turbulence model was applied. In addition, buoyancy was considered for analysis of the indoor airflow caused by heat gain from occupants and the resulting movement of airborne pathogens. The surface with incoming airflow through a ventilation system and windows was processed as the velocity inlet and the exhaust vent was processed as the pressure outlet.

**Table 2:** Boundary conditions

Type	All models
Physical conditions	Steady state Flow: segregated flow Energy: segregated fluid temperature Turbulence: k-e turbulence model Gas: air, ideal gas, incompressible Gravity: − 9.81 m/s Solver: steady state
Meshing conditions	Type: polyhedral mesh Prism layer thickness: 5% of the base (10 layers)
Room size	5 m × 5 m × 2.6 m
Inlet and outlet properties	Inlet: velocity inlet Outlet: pressure outlet
Inlet (outlet) area of room ventilation system	0.04 m^2^ × 4 each (0.16 m^2^)
Ventilation rate of room ventilation system	6 ACH (1069 CMH)
Operable windows and doors	Windows: 0.6 m × 1.6 m × 6 EA (4.8 m^2^) Doors: 1 m × 2.1 m × 2 EA (4.2 m^2^)
Airborne pathogen source	Exhalation: 15 m^3^/day (for teacher) Mouth size: 0.005 m^2^ Airborne pathogen concentration in exhalation: 1.0 (Passive scalar)
Thermal conditions	Inlet flow and walls: 300 K Human body and exhalation: 310 K
Convergence criteria	< 10^−4^

## Results

**Fig. 3:**
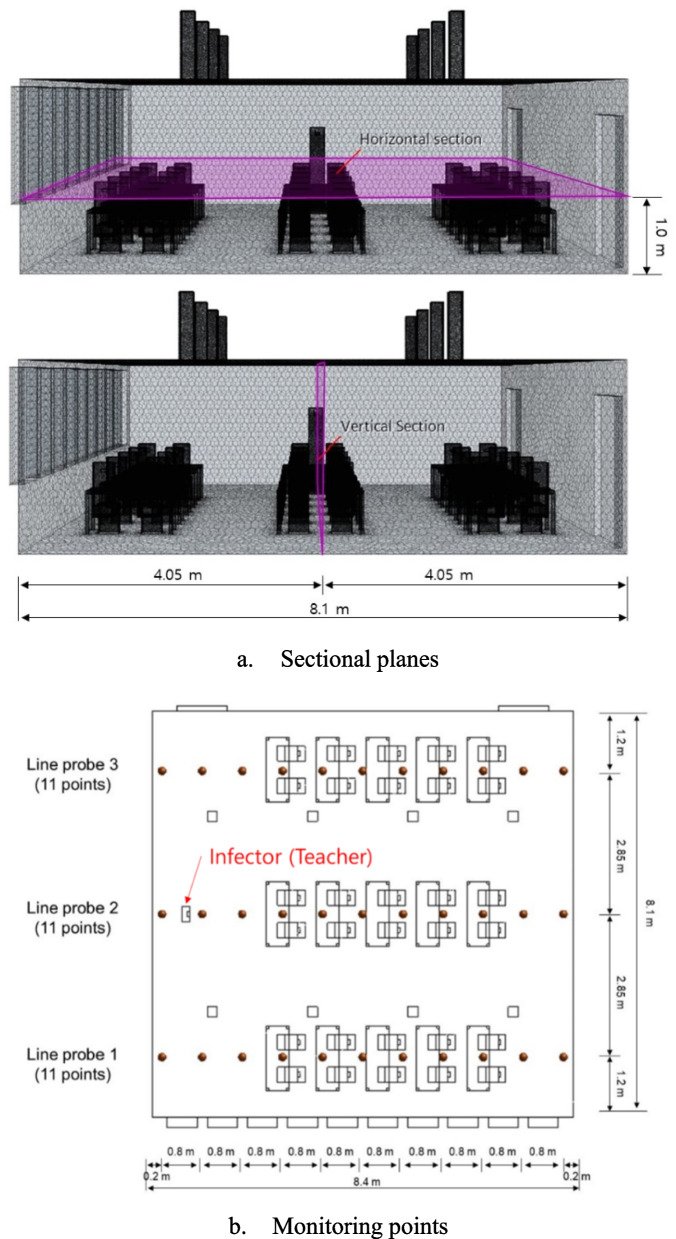
Monitoring sections for passive scalar concentrations a Sectional planes. b Monitoring points

**Fig. 4:**
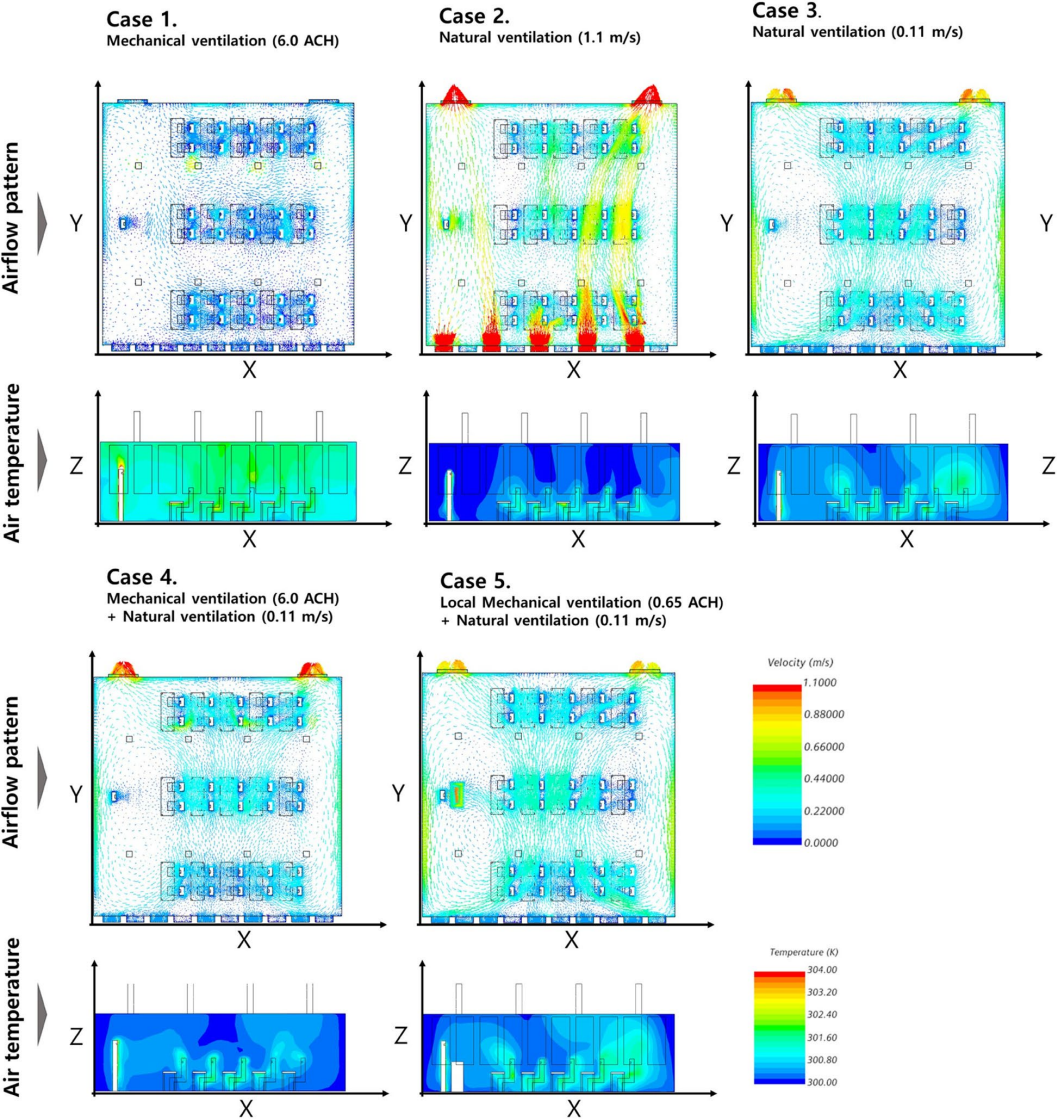
Airflow patterns (horizontal sections) and air temperature patterns (vertical sections)

**Fig. 5:**
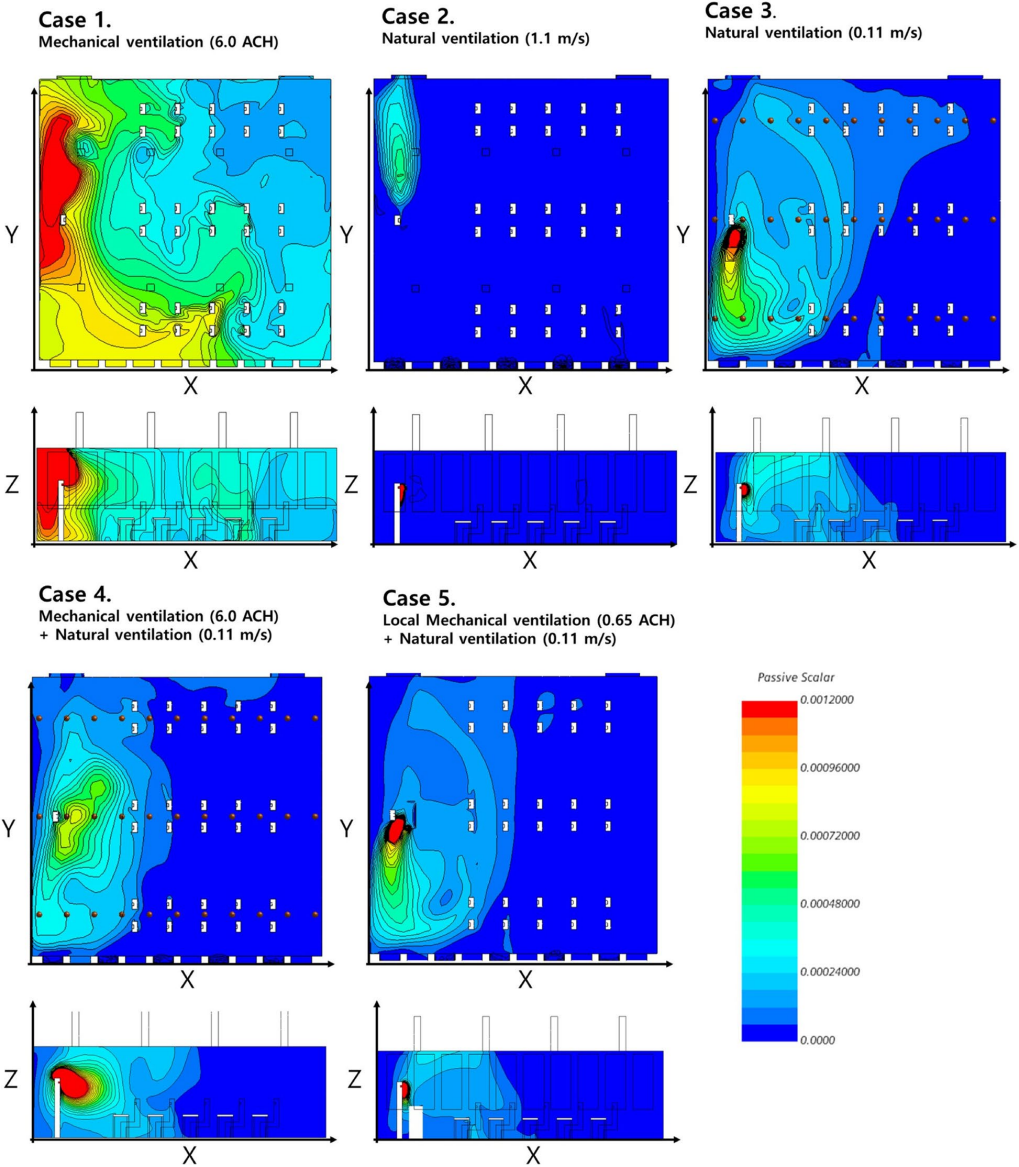
Passive scalar (airborne pathogen) concentration pattern at horizon h = 1.0 m and vertical section

**Fig. 6:**
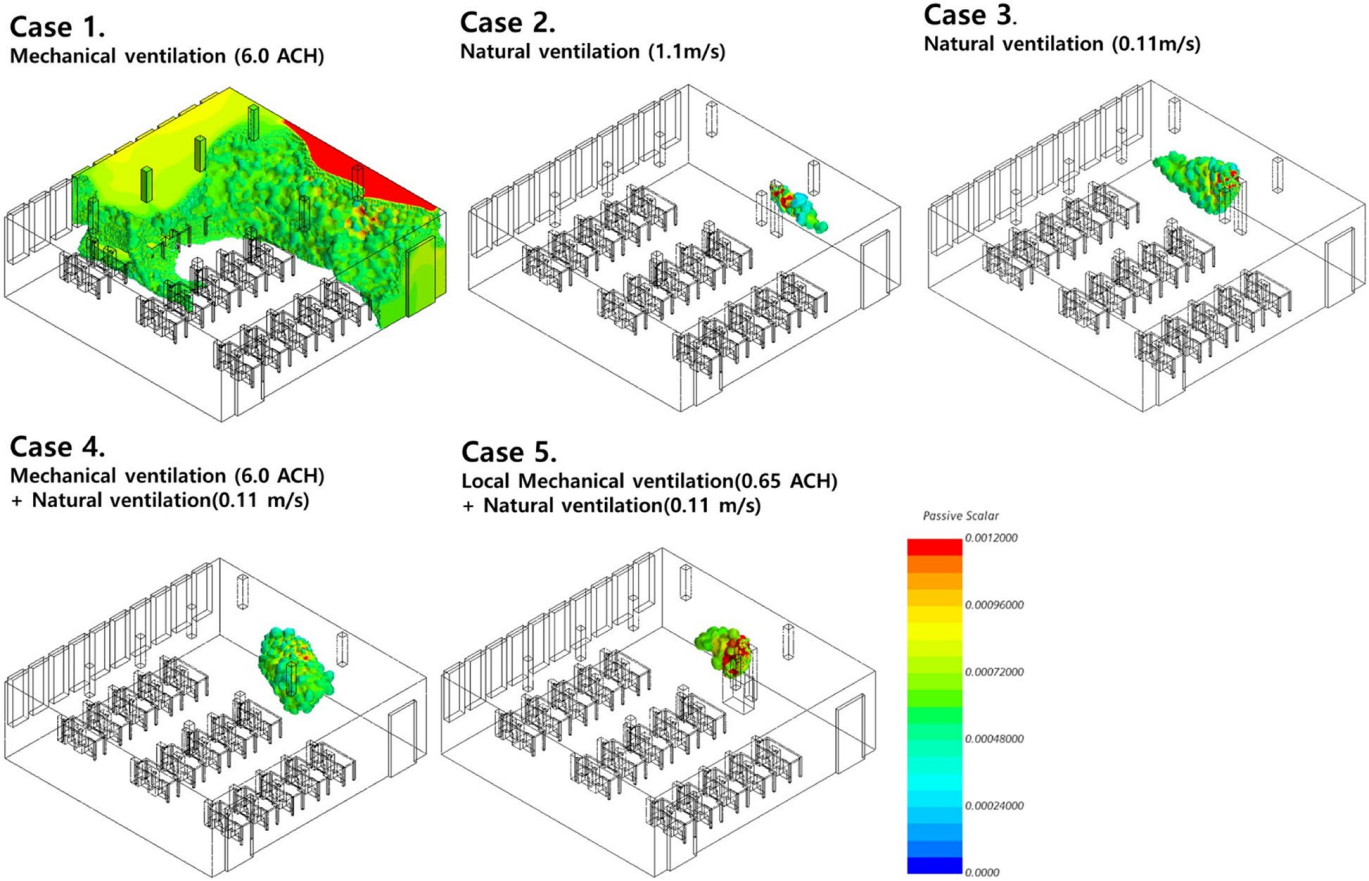
Passive scalar spread pattern of each case (passive scalar > 0.0006)

Figure 3 shows the monitoring sections for passive scalar concentrations. As presented in Fig. 4 the temperature distribution was based on the vertical sections in the center of the room, and the airflow distribution was based on the horizontal sections of the room. Figure 5 show the distribution of indoor passive scalar concentration. As presented in Fig. 6, the passive scalar was showed.

Case 1 represented the general mechanical ventilation condition in which indoor airflow velocity is limited and the temperature difference between the upper and lower parts of the classroom is also relatively small. In comparison, in Cases 2–5, the indoor temperature difference on the vertical sections was larger than in Case 1 due to the inflow of outdoor air. In addition, since the air temperature in the vicinity of the human body is higher than that of the surrounding area, air around the students actively ascended due to buoyancy.

In Case 1, a stable state was observed for the indoor airflow patterns due to the low airflow velocity. In Case 2, patterns of fast airflow from the window to the door on the opposite side were formed since the outdoor wind velocity at 1.1 m/s was relatively high under natural ventilation conditions. Case 3 represented the natural ventilation condition but because of the low incoming airflow velocity at 0.11 m/s, this case was strongly affected by the rising airflow due to heat gain from the students in the center of the classroom. Therefore, the incoming airflow from the window moved toward the door and passed through the center of the classroom. Due to these airflow patterns, the airflow vortices were formed close to the teacher, toward the window side. These patterns revealed the risk of pathogens from the teacher moving toward the airflow inlet thereby causing the dispersal of pathogens into the classroom. Case 4 combined natural ventilation at a low wind velocity of 0.11 m/s and general mechanical ventilation. Some interference occurred because of the opposing direction of the airflow for natural and mechanical ventilation. However, the overall airflow pattern was similar to that of other natural ventilation conditions. Case 5 added local ventilation on the teacher’s desk to the natural ventilation conditions described in Case 3. A relatively fast airflow pattern was formed in the vicinity of the exhaust vent of local ventilation.

Figure 5 shows the patterns of passive scalar concentrations on horizontal and vertical sections. Under the general mechanical ventilation conditions of Case 1, the passive scalar (airborne pathogens) discharged from the teacher are illustrated as spreading to the whole room. The spreading direction of the passive scalar coincided with the indoor airflow direction formed by the locations of air inlets and outlets of the mechanical ventilation system.

In Case 2, patterns showed that the passive scalar was rapidly discharged to the exhaust side by the fast inflow velocity of outdoor air. Therefore, the spreading of passive scalar to the inside of the classroom was limited.

Case 3 represented a condition of outdoor air flowing in at low velocity. In this condition, as seen in Fig. 2, the airflow vortices in the front of the classroom spread the passive scalar to the front of the classroom. This indicated that even if natural ventilation was carried out at the location of the elementary school teacher, when the incoming wind velocity is low, the possibility of students occupying the front of the classroom contracting airborne infections cannot be completely ruled out. Case 4 described a condition of combining natural ventilation with low wind velocity and conventional mechanical ventilation. The passive scalar concentration at the front of the classroom was slightly lower than that in Case 3. In Case 5, the passive scalar was rapidly removed by the local exhaust system installed on the teacher’s desk, and accordingly, the spreading of the passive scalar throughout the classroom by the vortices was also limited.

The effect of the local ventilation system on the teacher’s desk was clearly shown in the sections of Cases 3–5. Case 3 and Case 4 used a natural or conventional ceiling-mounted ventilation system. In these cases, airborne pathogens discharged from the teacher actively spread to the upper part of the classroom by buoyancy and indoor airflow patterns. Thus, airborne pathogens were relatively easily transmitted to the students seated in front of the teacher. However, in Case 5, students were exposed to relatively low concentrations of airborne pathogens because the airborne pathogens discharged from the teacher were immediately removed by the local exhaust system.

Figure 6 shows a 3D illustration of the movement patterns of airborne pathogens according to indoor airflow patterns. This is a representation of indoor spaces that shows more than an arbitrary passive scalar concentration.

In Case 1, the airborne pathogens discharged from the teacher moved to the exhaust vent on the window side installed on the ceiling of the classroom. The airborne pathogens that moved to the exhaust vent of the ventilation system were not sufficiently released outdoors but were recirculated back indoors. In addition to the presentation in Fig. 3, the airborne pathogens actively spread to the space in the upper part of the classroom through buoyancy.

**Fig. 7:**
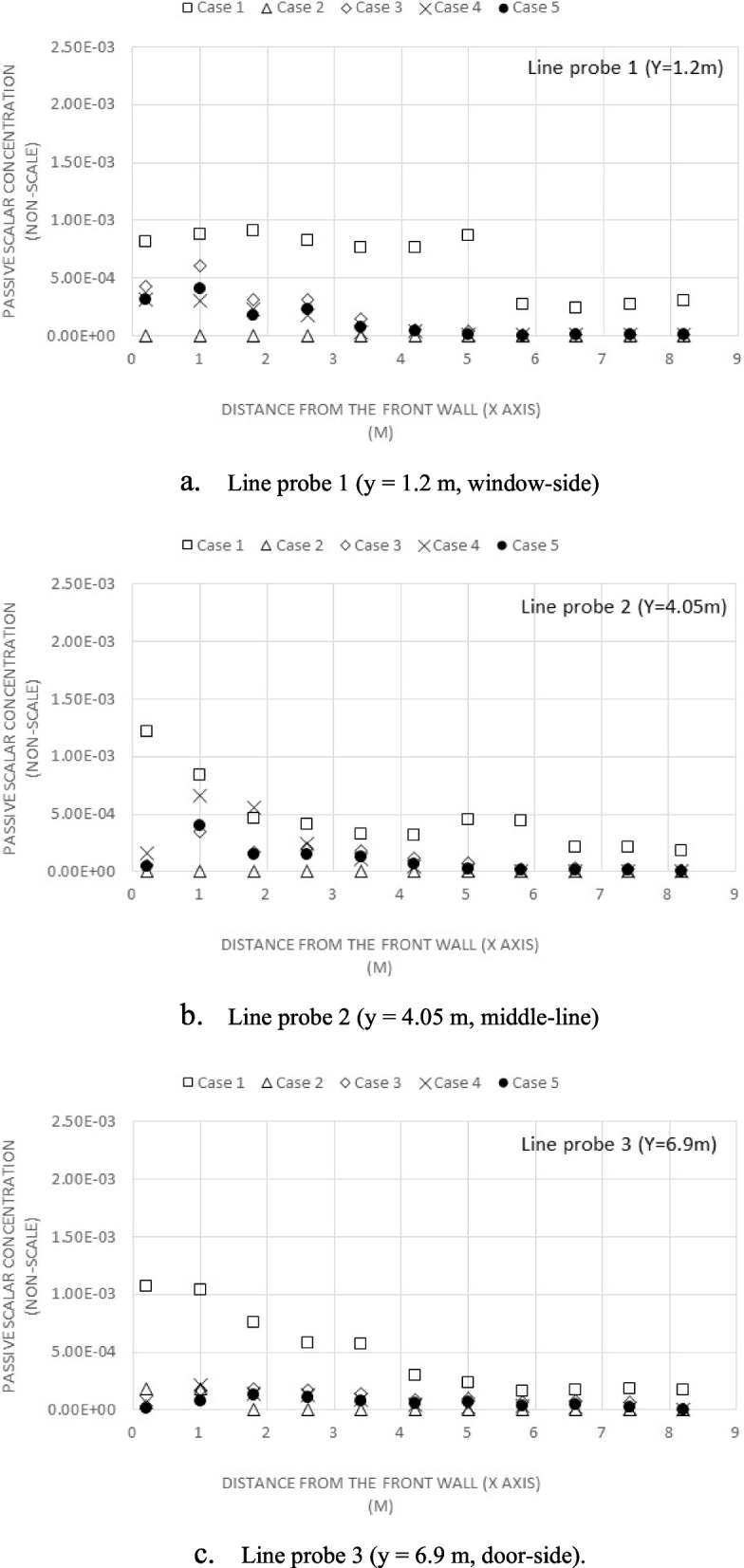
Passive scalar (airborne pathogen) concentrations at monitoring points (h = 1.0 m). **a** Line probe 1 (y = 1.2 m, window-side). **b** Line probe 2 (y = 4.05 m, middle-line). **c** Line probe 3 (y = 6.9 m, door-side)

In Case 2, the airborne pathogens were rapidly discharged toward the door on the opposite side of the classroom by the rapid air flowing in from the window side. This observation is in agreement with the 2D pattern result shown in Fig. 4. In Case 3, a natural ventilation condition with low wind velocity, and Case 4, a combination of natural and mechanical ventilation, had a greater spread of airborne pathogens toward the students in front of the classroom compared to the situation in Case 2. In Case 5, natural ventilation with low wind velocity was combined with local ventilation on the teacher’s desk. The airborne pathogens discharged from the teacher were rapidly removed from the teacher’s desk by local ventilation, effectively limiting the spread of airborne pathogens. Figure 7 showed the passive scalar spread pattern of each case.

Table 3 summarizes the average passive scalar concentrations in the horizontal plane of the height of the respiratory line and the whole classroom for each case. Because a higher ventilation rate could be obtained with natural ventilation conditions (Cases 2–5) than with mechanical ventilation conditions (Case 1), low passive scalar concentrations were seen in the horizontal plane on the respiratory line and in the whole room compared to the concentration under mechanical ventilation condition (Case 1). The results demonstrated the advantage of natural ventilation in the control of airborne pathogens, and that the effect was proportional to the ventilation rate.

**Table 3:** Average passive scalar concentration at breathing zone and whole room (non-scale)

Category		Case 1	Case 2	Case 3	Case 4	Case 5
Breathing zone (Horizontal plane 1 m above floor)	Average concentration	5.05 E–04	1.61 E–05	1.24 E–04	1.09 E–04	9.07 E–05
	Reductions	0.0%	96.8%	75.4%	78.4%	82.0%
Entire room	Average concentration	5.35 E–04	1.03 E–05	1.11E–04	1.12 E–04	7.58 E–05
	Reductions	0.0%	98.1%	79.3%	79.1%	85.8%

While Table 3 shows the average passive scalar concentration at the height of the respiratory line and the entire room, Fig. 7 shows the distribution of local passive scalar concentrations in the vicinity of the occupants. In Fig. 7, the position X = 0 indicates the wall surface in front of the classroom.

Case 1, the mechanical ventilation condition, showed high values for the overall passive scalar concentrations for most of the monitoring points. Case 2, the natural ventilation condition with the maximum airflow rate, showed the lowest values. Case 4 and Case 5 could lower the passive scalar concentrations compared to Case 3. However, in Case 3 and Case 4, where the air inflow rate was low (0.11 m/s) during natural ventilation, the passive scalar concentration in the front part of the classroom where the teacher was standing (X < 4 m) was higher than in the back of the classroom. This was particularly noticeable in the front area of the classroom with vortices formed by buoyancy in the center of the classroom under low wind velocity conditions (see Fig. 7a, b). In Case 5, the passive scalar concentration in the area could be significantly reduced owing to the application of the local exhaust system with a relatively lower ventilation rate than the mechanical ventilation system for the entire room of Case 4 (Fig. 7a).

## Discussion

The analysis confirmed that the overall ventilation rate in the classroom had a direct impact on reducing the concentrations of airborne pathogens (passive scalar) regardless of the type of ventilation system. In general, rather than the condition with the application of mechanical ventilation alone (Case 1), the condition with sufficient utilization of natural ventilation (Cases 2 and 3) or a condition with a combined application of natural ventilation and mechanical ventilation (Cases 4 and 5) was more effective in removing and controlling airborne pathogens.

It should be noted that while natural ventilation has the advantage of allowing air inflow to the indoor space, the ventilation rate is limited when the outdoor wind velocity is low. However, the results showed that even in the case of low wind velocity (defined as 1/20 of the regional average wind velocity), a higher ventilation rate was obtained than for the mechanical ventilation condition (6 ACH). The possibility of obtaining lower ventilation rates due to even lower outdoor wind velocity and insufficient operable window area still exists. Therefore, the continuous operation of the mechanical ventilation system was necessary to ensure the minimum ventilation rate was met and could compensate for the uncertainty of the natural ventilation system.

Furthermore, the simulation results showed that for low airflow velocity introduced through the windows, the possibility existed that airborne pathogens discharged from the teacher in the front of the classroom might spread to the entire classroom space through airflow vortices. This was because the indoor buoyancy by occupants had a greater impact on the indoor airflow patterns than the outdoor wind velocity, especially when the outdoor wind velocity was low. If the outdoor wind velocity was zero or lower than the simulated conditions, the source of infection generated by the teacher slowly moved toward the students due to the buoyancy of the air around the students. The Case 5 simulation showed that applying a local ventilation system between the teacher and the students was able to control the spread of airborne pathogens caused by vortices in the front area of the classroom effectively with low ventilation rate (0.65 ACH).

## Conclusion

This study analyzes the patterns of the spread of airborne pathogens under different ventilation conditions to establish countermeasures to prevent the spread of airborne pathogens discharged from teachers. These conditions include general mechanical ventilation, natural ventilation, hybrid general ventilation, and hybrid local ventilation.

The results of this study can be outlined as follows:

Regardless of the ventilation method applied in the classroom, an increase in the ventilation rate can lower the concentration of airborne pathogens for the entire space of the classroom. The natural ventilation conditions with high wind velocity are confirmed to be effective in removing airborne pathogens.

The application of the hybrid ventilation system is confirmed to be effective as a method for preventing the spread of airborne pathogens by increasing the indoor ventilation rate. The hybrid ventilation which combines mechanical and natural ventilation performed the highest among the simulation models and features the advantages of both methods. This includes the high ventilation rate advantage of the natural ventilation system and the consistency of the mechanical ventilation in securing the minimum ventilation rate required.

The present study shows that the spread of airborne pathogens caused by vortices can be effectively prevented through the application of a local ventilation system. Under natural ventilation conditions with low wind velocity, ascending airflow occurs around occupants in the center of the classroom, causing vortices and therefore the movement of air from the window to the central part of the classroom. At the teacher, who is assumed to be the infected person in this study, vortices can be formed in which air flows from the exhaust vent (door) to the air inlet (window). This results in the spreading of airborne pathogens to students. Therefore, installing a local ventilation system close to the infected person may control this dispersal pathway and protect student from exposure to pathogens unwittingly spread by teachers.

During the COVID-19 pandemic, elementary schools have experienced difficulties in infection management in school environments. South Korea has experienced a renewed spread of infections due to the implementation of face-to-face attendance in schools. This study provides basic reference data on the efficacy of different types of ventilation systems to control the spread of airborne pathogens in classrooms. The findings of this study present guidelines for the future improvement of safety at educational facilities like classrooms for teachers and students alike.

## Data Availability

The datasets generated during the current study are not publicly available due to approval of grant but are available from the corresponding author on reasonable request.
